# Analytical Evaluation of the New Beckman Coulter Access Procalcitonin (PCT) Chemiluminescent Immunoassay

**DOI:** 10.3390/diagnostics10030128

**Published:** 2020-02-26

**Authors:** Giuseppe Lippi, Gian Luca Salvagno, Matteo Gelati, Mairi Pucci, Davide Demonte, Diego Faggian, Mario Plebani

**Affiliations:** 1 Section of Clinical Biochemistry, University of Verona, 37134 Verona, Italy; giuseppe.lippi@univr.it (G.L.); matteo.gelati@univr.it (M.G.); mairipucci@yahoo.it (M.P.); davide.demonte@studenti.univr.it (D.D.); 2 Department of Laboratory Medicine, University Hospital of Padova, 35128 Padova, Italy; diego.faggian@aopd.veneto.it (D.F.); mario.plebani@unipd.it (M.P.)

**Keywords:** sepsis, infection, procalcitonin, immunoassay

## Abstract

This study was designed to evaluate the analytical performance of the recently commercialized Beckman Coulter Access procalcitonin (PCT) chemiluminescent test on the Access immunoassay system. The analytical assessment encompassed the estimation of limit of blank (LoB), limit of detection (LoD), functional sensitivity (i.e., PCT value with ≤10% imprecision), linearity, imprecision and comparability of values with BRAHMS PCT-sensitive Kryptor. LoB, LoD and functional sensitivity were 0.002 μg/L, 0.003 μg/L and 0.003 μg/L, respectively. Intra-assay, inter-assay and total imprecision for plasma pools with low, medium and high PCT values were 1.8–2.1%, 2.4–3.7% and 3.1–4.3%, respectively. The assay exhibited excellent linearity between 0.02 and 84.0 μg/L. Excellent correlation (r = 0.999; *p* < 0.001) and negligible bias (3.2%) were found by comparing values obtained in paired plasma samples with BRAHMS PCT-sensitive Kryptor. Diagnostic agreement at 0.5, 2.0 and 10 μg/L PCT values ranged between 98%-100%. The results of this study confirm that Access PCT displays excellent analytical performance and high comparability with BRAHMS PCT-sensitive Kryptor.

## 1. Introduction

Procalcitonin (PCT) is the 116 amino acid precursor of the active hormone calcitonin, which is almost uniquely synthesized by thyroid C cells under physiological conditions, and displays plasma concentrations <0.05 μg/L in healthy subjects [1,2]. In response to proinflammatory triggers, especially bacterial products (i.e., endotoxin), a sustained extra-thyroid production occurs, mostly from the leukocytes, liver, lung, intestine and kidney, thus enhancing its circulating levels by several orders of magnitude, occasionally over 100 μg/L [1,2,3]. This paradigmatic biological event, which was first documented by Wagner and colleagues in 1981 [4], was then exploited by Marcel Assicot et al. for demonstrating that measuring PCT may be a valuable aid for diagnosing severe bacterial infections [5].

Since the publication of Assicot’s study over 25 years ago [5], a large volume of clinical evidence has been made available, indicating that serial PCT assessment provides highly clinically useful information for diagnosing infectious diseases, but also for guiding antimicrobial therapy, thus contributing to improvement of clinical outcomes, lowering healthcare expenditures [6,7,8,9], and potentially mitigating the burden of antibiotic resistance, which is one of the major worldwide threats as emphasized by the World Health Organization (WHO, Geneva, Switzerland) [10]. Some decisional cut-offs are currently being proposed, namely <0.25 μg/L for ruling out bacterial infection, >0.5 μg/L for diagnosing local bacterial infection, >2.0 μg/L for diagnosing systemic infection (i.e., sepsis), and >10 μg/L for diagnosing severe sepsis and/or septic shock [1,2].

The well-established value of PCT measurement in many infectious conditions and across a kaleidoscope of healthcare settings has strongly contributed to development of analytical techniques for its measurement [11]. More specifically, a large number of fully-automated immunoassays have become commercially available during the past decade, employing different assay formats (i.e., enzymatic, luminescent, fluorescent, chemiluminescent and turbidimetric immunoassays) [12,13,14,15,16,17,18,19]. Since the clinical management of patients with severe infections and/or sepsis is always critical and time-dependent, the availability of fully-automated PCT immunoassays is now virtually unavoidable, and these techniques enable an excellent balance between high throughput, short turnaround time, low sample volume (which is always crucial in critical patients needing repeated blood collections) and reasonable costs. This study was hence designed to evaluate the analytical performance of the recently commercialized Beckman Coulter Access PCT chemiluminescent immunoassay and comparing the results of this method with those of a well-established (reference) technique.

## 2. Materials and Methods

### 2.1. Description of Access PCT Immunoassay

The Access PCT is a paramagnetic particle chemiluminescent immunoassay developed by Beckman Coulter (Brea, CA, USA) for quantitative assessment of PCT on the family of Access immunoassay systems. Briefly, in this two-step sandwich immunoassay, anti-PCT monoclonal antibodies conjugated with alkaline phosphatase are added to the reaction cuvette along with a patient sample and reagent buffer. After short incubation time, paramagnetic particles coated with monoclonal anti-PCT antibodies are added to the test mixture. PCT binds to anti-PCT antibodies on a solid phase, whilst anti-PCT antibodies conjugated with alkaline phosphatase react with different antigenic sites on PCT molecules. After incubation, the antigens bound to the solid phase are held in a magnetic field, whilst the unbound material is washed and discarded. The chemiluminescent substrate is then added to the cuvette and the light generated by this reaction is measured with a luminometer. Photon generation is directly proportional to PCT concentration in the test sample, and the final value of the analyte is calculated using a multi-point calibration curve.

The method is calibrated against a proprietary standard (PN C22594) and does not use BRAHMS antibodies. Regarding the other characteristics, serum, lithium, heparin, and ethylenediaminetetraacetic acid (EDTA) plasma can be used. The sample volume is 35 μL, and the time to the first result is ~20 min. According to the manufacturer’s declarations, the measurement range is between 0.01 and 100 μg/L (up to 1000 μg/L with the special dilution feature), the limit of blank (LoB) is 0.001 μg/L, the limit of detection (LoD) is 0.002 μg/L, the functional sensitivity (defined as value with imprecision ≤20%) is 0.002 μg/L, whilst the total imprecision is ≤8.0% for a PCT concentration of 0.15 μg/L. The open reagent pack and the calibration curve are stable for 42 days, whilst the open calibrator vial is stable for 90 days.

### 2.2. Evaluation of the Analytical Performance of Access PCT

The evaluation of the analytical performance of Beckman Coulter Access PCT on a Beckman Coulter Access immunoassay system encompassed the estimation of LoB, LoD and functional sensitivity. The LoB was estimated as the sum of mean values and of (1.645 × standard deviation (SD)), obtained from ten consecutive replicate measures of saline, as described elsewhere [20]. The LoD was estimated as the sum of LoB and of (1.645 × SD) of ten replicate measures of a patient plasma sample displaying the lowest measurable PCT value (i.e., ~0.001 µg/L) [20]. The functional sensitivity was finally calculated as the lowest measurable PCT with imprecision (i.e., coefficient of variation, CV) ≤10%. This value was calculated by serially diluting a patient plasma sample displaying a PCT concentration of ~0.25 μg/L with saline (dilutions ranged between 1:2 and 1:256). All sample dilutions were then assayed in ten consecutive replicate runs and imprecision was calculated for each dilution. A model fit was then developed for extrapolating the PCT value with imprecision ≤10%.

### 2.3. Imprecision Studies

The imprecision of Access PCT was assessed using three plasma pools exhibiting low (i.e., 0.29 μg/L), intermediate (i.e., 2.83 μg/L) and high (i.e., 11.83 μg/L) PCT values, respectively. All plasma pools were obtained by pooling ten anonymized plasma samples previously collected into evacuated, lithium-heparin blood tubes (3.5 mL, 13 mm × 75 mm, lithium-heparin plus gel, reference n. 12554; Vacutest Kima, Padova, Italy), and conveyed to the laboratory of the University Hospital of Verona for routine PCT assessment. The pools were prepared by pooling identical volumes of ten different patient samples which were appropriately mixed, divided into identical aliquots of ~1.5 mL and immediately frozen at −70° C. Intra-assay imprecision was assayed by 20 sequential measurements of a single aliquot of each pool, whilst inter-assay imprecision was assayed by thawing one frozen aliquot of each pool and measuring PCT in duplicate for 10 consecutive working days. Total imprecision was calculated using the formula of Krouwer and Rabinowitz [20].

### 2.4. Linearity

A routine plasma sample displaying a high PCT value (i.e., 84.0 μg/L) was serially diluted at fixed ratios (1:9; 2:8; 3:7; 4:6; 5:5; 6:4; 7:3; 8:2; 9:1) along with another routine plasma sample with a very low PCT concentration (i.e., 0.02 μg/L), thus covering the more clinically significant range of PCT values in health and disease. Each serial dilution was tested in duplicate and theoretical values were calculated from measured values of undiluted specimens and plotted into a diagram. Linearity was finally tested by calculating Pearson’s correlation coefficient (r).

### 2.5. Method Comparison

The comparison studies were performed using all consecutive anonymized lithium-heparin plasma samples referred to the local laboratory over one working day for routine PCT testing (n = 213). Samples were divided in identical aliquots and assessed with Access PCT and with the reference technique BRAHMS PCT-sensitive Kryptor (Thermo Fisher Scientific, Waltham, MA, USA), which has historically been used for setting the clinically validated PCT decision thresholds [21]. This second test is a homogeneous immunoassay based on the sandwich principle and using TRACE (time resolved amplified cryptate emission) technology. According to the manufacturer’s specifications, the functional sensitivity of BRAHMS PCT-sensitive Kryptor is 0.06 μg/L, the assay is linear between 0.02 and 5000 μg/L with automatic dilution (direct measuring range is 0.02–50 μg/L), whilst inter- and intra-assay imprecision was recently reported as being between 1.66–3.28% and 0–0.46%, respectively [16]. The main characteristics of the two assays (BRAHMS PCT-sensitive Kryptor vs. Beckman Coulter Access PCT) are as follows: type of assay: homogeneous immunoassay using TRACE technology vs. paramagnetic, chemiluminescent immunoassay; incubation time: 19 vs. 20 min; calibration stability: 15 vs. 42 days; throughput: up to 155 vs. up to 400 tests per hour (with UniCel DxI 800). The correlation between methods was assessed with Passing and Bablok regression and Spearman’s correlation coefficient (r), whilst the mean bias (with 95% confidence interval (95% CI)) was calculated using Bland–Altman plot analysis. The agreement (and kappa statistics) of values were assessed at the three more significant PCT diagnostic thresholds (i.e., 0.5, 2.0 and 10 μg/L). The full list of interfering substances is available in the methods manuals.

### 2.6. Statistics and Ethics Committee Approval

The statistical analysis was carried out using Analyse-it (Version 2.30; Analyse-it Software Ltd., Leeds, UK), setting statistical significance at *p* < 0.05. The study was carried out by employing pre-existing plasma samples for which routine PCT assessment was already requested, so that patients’ informed consent was unnecessary. Data obtained on Access PCT were not reported and did not impact clinical management. The study was approved by the Ethics Committee of the University Hospital of Verona (SOPAV2; 25 July 2016).

## 3. Results

### 3.1. Analytical Performance

The LoB and LoD of Access PCT, calculated as described in the previous section of this article, were 0.002 μg/L and 0.003 μg/L, respectively. The 10% imprecision of the assay was found to correspond to a PCT concentration of 0.003 μg/L, thus almost aligned with manufacturer’s declarations (i.e., 0.002 μg/L for imprecision ≤20%).

### 3.2. Imprecision Studies

The results of imprecision studies are summarized in Table 1. The intra-assay and inter-assay imprecision for plasma pools with low, medium and high PCT values was between 1.8% and 2.1% and between 2.4% and 3.7%, respectively. The total analytical imprecision was instead between 3.1% and 4.3%.

### 3.3. Linearity Studies

The Access PCT immunoassay exhibited an excellent linearity throughout a significant range of PCT concentrations in health and disease (0.02–84.0 μg/L). The equation of Passing and Bablok regression analysis was *y* = 1.05*x*−1.37, whilst Spearman’s correlation coefficient was 0.999 (95% CI, 0.997–1.000; *p* < 0.001).

### 3.4. Method Comparison

**Figure 1:**
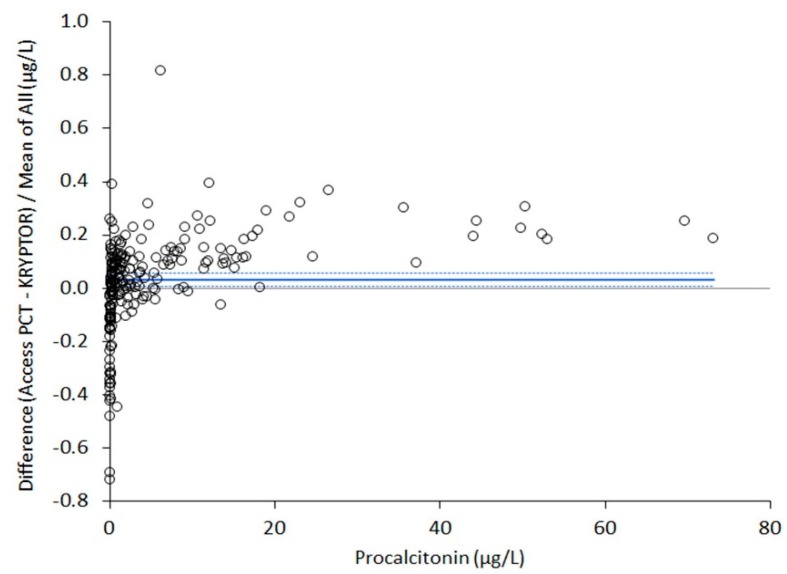
Bland–Altman plot analysis of procalcitonin (PCT) values obtained with Beckman Coulter Access PCT on the Access immunoassay system and BRAHMS PCT-sensitive Kryptor. The straight line defines the mean bias, whilst the dotted lines are drawn at the 95% confidence interval.

Five out of the 213 plasma samples originally collected for this study were excluded for the presence of haemolysis (i.e., plasma haemoglobin >0.5 g/L). Therefore, the final sample size consisted of 208 plasma aliquots. An excellent correlation was found between Access PCT and BRAHMS PCT-sensitive Kryptor. In particular, Spearman’s correlation coefficient was 0.999 (95% CI, 0.997–0.998; *p* < 0.001), whilst the equation of Passing and Bablok regression was [Access PCT] = 1.12*x* [Kryptor PCT] − 0.03. The results of Bland–Altman plot analysis are shown in Figure 1. The mean percentage bias of Access PCT versus Kryptor PCT was 3.2% (95% CI, 0.7–5.7%). The diagnostic agreement of the two immunoassays at the three clinically significant diagnostic thresholds was 100% (kappa statistic, 1.00; 95% CI 1.00–1.00) at 0.5 μg/L, 98% (kappa statistic, 0.97; 95% CI, 0.94–1.00) at 2.0 μg/L and 98% (kappa statistics, 0.91; 95% CI, 0.84–0.99) at 10 μg/L, respectively.

## 4. Discussion

The number of PCT immunoassays available in the market is constantly increasing in parallel with the many and multifaceted diagnostic applications of this biomarker [22,23]. A comprehensive validation of the analytical performance of any new test thus represents a necessary premise before allowing its widespread clinical usage [24], and PCT immunoassays make no exception to this rule.

The results of our analytical evaluation of the newly commercialized Beckman Coulter Access PCT chemiluminescent immunoassay demonstrate the reliability and robustness of this fully automated technique. The values of LoB, LoD and functional sensitivity were considerably low (all ≤0.003 µg/L), in line with those claimed by the manufacturer and aligned with those of the most sensitive chemiluminescent immunoassays such as Fujirebio Lumipulse BRAHMS PCT [14], but were even better than those of other fully-automated techniques [13,17], including the currently considered reference method BRAHMS PCT-sensitive Kryptor [16]. The total imprecision of the assay was also globally acceptable, being between 3.1% and 4.3% and thus mirroring the performance characteristics of other comparable fully automated chemiluminescent immunoassays [13,14,17]. The linearity range assessed in our study was also satisfactory, covering the more significant range of PCT concentrations in health and disease. The results of the comparison study with BRAHMS PCT-sensitive Kryptor were especially surprising and favorable. Overall, the correlation coefficient was excellent (i.e., r = 0.999; *p* < 0.001) and the bias extremely limited, lower than 5% (i.e., 3.2%), and thus even better than those frequently observed when comparing other methods using the same BRAHMS anti-PCT monoclonal antibodies [12,13,16,17]. Identical conclusions can be made for diagnostic agreement at the three clinical decision thresholds, whereby global agreement between Access PCT and BRAHMS PCT-sensitive Kryptor was always ≥98%, again, better than comparative methods based on BRAHMS anti-PCT monoclonal antibodies.

In conclusion, low sample volume (i.e., 35 μL), rapid turnaround time (i.e., ~20 min) and high throughput enabled by adaptation to multiple systems belonging to the Beckman Coulter immunoassay family, combined with excellent analytical performance and high comparability with the reference BRAHMS PCT-sensitive Kryptor technique, would allow us to conclude that the new Beckman Coulter Access PCT chemiluminescent immunoassay is an optimal alternative for routine and urgent assessment of PCT in clinical practice.

## Figures and Tables

**Table 1:** Analytical imprecision of the Beckman Coulter Access procalcitonin (PCT) immunoassay.

Pools	Intra-Assay				Inter-Assay				Total
	n	Mean (µg/L)	SD (µg/L)	CV	n	Mean (µg/L)	SD (µg/L)	CV	CV
Pool low	20	0.29	0.01	1.8%	10	0.30	0.01	3.6%	4.0%
Pool medium	20	2.83	0.05	1.9%	10	2.85	0.07	2.4%	3.1%
Pool high	20	11.23	0.23	2.1%	10	10.92	0.41	3.7%	4.3%

SD, standard deviation; CV, coefficient of variation.
